# Increased lipopolysaccharide content is positively correlated with glucocorticoid receptor‐beta expression in chronic rhinosinusitis with nasal polyps

**DOI:** 10.1002/iid3.346

**Published:** 2020-09-01

**Authors:** Shui‐Bin Wang, Shi‐Ming Chen, Ke‐Sheng Zhu, Bin Zhou, Long Chen, Xiao‐Yan Zou

**Affiliations:** ^1^ Department of Otolaryngology—Head and Neck Surgery Yichang Yiling Hospital Yichang China; ^2^ Department of Otolaryngology—Head and Neck Surgery Renmin Hospital of Wuhan University Wuhan China; ^3^ Department of Laboratory Medicine Yichang Yiling Hospital Yichang China

**Keywords:** chronic rhinosinusitis with nasal polyps, *Escherichia coli*, lipopolysaccharide

## Abstract

**Introduction:**

Chronic rhinosinusitis with nasal polyps (CRSwNP) is a common and frequently occurring disease of the upper respiratory tract. The nasal instillation of the Gram‐negative (G^−^) bacterial product lipopolysaccharide (LPS) can induce not only acute sinusitis but also the development of CRSwNP in animal models. Nevertheless, the expression and distribution of LPS in patients with CRSwNP have not been investigated. And the study was to investigate the expression of LPS and its relationship with glucocorticoid receptors (GRs) in CRSwNP.

**Methods:**

Multiple methods, including bacterial culture and immunohistochemistry, were used to detect and analyze nasal bacteria, plasma LPS content, and the levels of LPS and GR‐α/β, cluster of differentiation 68 (CD68), and myeloperoxidase (MPO) expression, as well as their relationship in CRSwNP.

**Results:**

The number of G^−^ bacteria and *Escherichia coli* (*E. coli*) was not significantly different between CRSwNP subjects and the controls. However, the positive rate of LPS was much higher than that of *E. coli* in CRSwNP subjects and was significantly higher in noneosinophilic CRSwNP subjects than in eosinophilic CRSwNP subjects. Moreover, the LPS levels were positively correlated with GR‐β but not GR‐α expression in CRSwNP. Immunofluorescence assays showed that LPS was mainly detected in CD68^+^ macrophages and MPO^+^ neutrophils, in addition to histiocytes, in CRSwNP.

**Conclusions:**

Persistent LPS in CRSwNP can lead to unresolved mucosal inflammation, eventually leading to tissue remodeling and the development of CRSwNP. Our findings suggest that increased LPS content and possible resistance to glucocorticoids may be one of the important pathogenic mechanisms of G^−^ bacteria in CRSwNP.

## Graphical abstract

We used bacterial culture and immunohistochemistry to find that the positive rate of lipopolysaccharide (LPS) was much higher than that of *Escherichia coli* in chronic rhinosinusitis with nasal polyps (CRSwNP), and the LPS levels were positively correlated with glucocorticoid receptor‐β (GR‐β) expression in CRSwNP. Our findings suggest increased LPS content and possible resistance to glucocorticoids may be one of the important pathogenic mechanisms of G‐negative bacteria in CRSwNP.

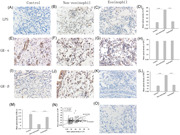

**Abbreviations**
CRSwNP: chronic rhinosinusitis with nasal polyps
*E. coli*: *Escherichia coli*
G^−^: Gram‐negative
G^+^: Gram‐positive
GR‐α: glucocorticoid receptor‐α
GR‐β: glucocorticoid receptor‐β
LPS: lipopolysaccharide
TLR4: Toll‐like receptor 4

## 1 INTRODUCTION

Chronic rhinosinusitis with nasal polyps (CRSwNP) characterized by chronic inflammation of the nasal cavity and paranasal sinus mucosa remains a significant health problem with a considerable socioeconomic burden and is increasing in prevalence and incidence. CRSwNP can be divided into eosinophilic and noneosinophilic types according to the degree of infiltrative eosinophils.^1^, ^2^, ^3^, ^4^ At present, the pathogenesis of CRSwNP remains unclear. Many studies support the hypothesis that allergens, bacteria, fungal infections, and nasal anatomic abnormalities all play an important role. Although the widespread use of intranasal glucocorticoids and endoscopic sinus surgery has significantly improved the cure rate of CRSwNP, approximately 10% of CRSwNP cases still have postoperative recurrent attacks.^5^, ^6^, ^7^ Therefore, the internal mechanisms of CRSwNP require further elucidation.

Glucocorticoid resistance is an important factor for the recurrence of CRSwNP.^6^, ^8^, ^9^, ^10^ Two subtypes of glucocorticoid receptor α/β (GR‐α/β) exist in CRSwNP. Exogenous glucocorticoids exert anti‐inflammatory and immune‐inhibitory effects mainly through GR‐α, which can be antagonized by GR‐β. Some studies suggest that glucocorticoid resistance is related to the upregulation of GR‐β or dysfunction of GR‐α in CRSwNP ^6^, ^9^, ^11^, ^12^

Noneosinophilic CRSwNP, mainly dominated by neutrophils, is closely related to bacterial infection, especially by Gram‐negative (G^−^) bacteria.^7^, ^13^, ^14^ Lipopolysaccharide (LPS), one of the principal chemical components of bacterial endotoxin, is located in the outer layer of the G^−^ bacterial cell wall and consists of three parts: lipid A, core polysaccharide, and O‐specific polysaccharide. In addition to its toxic effects, LPS can induce an immune response.^13^, ^15^, ^16^, ^17^, ^18^ Purified LPS is a noninfectious inflammatory factor and can regulate the release of inflammatory mediators, resulting in chronic rhinosinusitis.^15^ In animal models, instilling LPS into the nasal cavity of rats or mice can induce not only acute sinusitis but also the development of CRSwNP and olfactory dysfunction.^13^, ^19^, ^20^ These findings further confirmed that noninfectious inflammatory factors were involved in the occurrence and development of inflammatory diseases. Although previous studies have shown that a certain amount of G^−^ bacteria are present in CRSwNP, the expression and distribution of LPS in CRSwNP have not been investigated. On the basis of the effects of LPS and the results of the aforementioned studies, the purpose of this study is to investigate LPS expression and its possible relationship with GRs in CRSwNP and to further reveal the role and possible mechanisms of G^−^ bacteria in the development of CRSwNP.

## 2 MATERIALS AND METHODS

### 2.1 Subjects and samples

This study was approved by the Ethics Committee of Yichang Yiling Hospital. Informed consent was obtained from every subject. Samples were taken from patients undergoing endoscopic nasal surgery at the Department of Otolaryngology—Head and Neck Surgery of Yichang Yiling Hospital between July 2015 and July 2017, including 112 patients with CRSwNP and 50 patients with the deviated nasal septum (control group). The clinical data of the patients are summarized in Table 1. The diagnosis of CRSwNP was made according to the recommended European diagnostic standard, EPOS2012.^21^ Patients were excluded if they had a history of autoimmune disease, the “aspirin triad,” primary cilia motility dysfunction, cystic fibrosis, fungal CRSwNP, bronchopneumonia, inflammatory bowel disease, or hepatitis or had a history of intranasal or oral corticosteroid use in the 2 weeks before the surgery. During the surgery, specimens were taken from CRSwNP, and the inferior turbinate mucosa was collected from patients with nasal septum deviation. The specimens were fixed in 4% paraformaldehyde for 24 hours before embedding in paraffin for immunohistochemical studies.

**Table 1:** Subjects’ characteristics

Subjects	Control			Noneosinophilic CRSwNP			Eosinophilic CRSwNP		
Total number of subjects (male subjects)	50 (26)			63 (37)			49 (28)		
Age, y; median (range)	36 (16‐63)			44 (13‐71)			46 (19‐65)		
	Y	N	U	Y	N	U	Y	N	U
Atopy	0	50	0	1	62	0	4	42	3
Asthma	0	50	0	3	59	1	5	42	2

Samples	Turbinate			CRSwNP			CRSwNP		
Bacteria in the nasal cavity, the total number of subjects (male subjects)	50 (26)			63 (37)			49 (28)		
LPS in blood, the total number of subjects (male subjects)	50 (26)			63 (37)			49 (28)		
Immunohistochemistry, the total number of subjects (male subjects)	50 (26)			63 (37)			49 (28)		
Immunofluorescence, the total number of subjects (male subjects)	15 (8)			15 (12)			15 (6)		
Age, y; median (range)	46 (23‐58)			47 (23‐65)			46 (23‐65)		

*Note*: Values are presented as medians (ranges), where shown.

Abbreviations: CRSwNP, chronic rhinosinusitis with nasal polyps; LPS, lipopolysaccharide; N, No; U, unknown; Y, yes.

### 2.2 Haematoxylin and eosin staining of paraffin sections

Serial paraffin sections (4 µm) were subjected to haematoxylin and eosin staining in accordance with the manufacturer's protocols. CRSwNP was further classified into eosinophilic and noneosinophilic subtypes according to previously described methods.^1^

### 2.3 Bacterial culture of nasal secretions

Nasal secretions were collected with a sterile swab before surgery under a nasal endoscope. Bacteriological culture was performed according to a previously described method.^22^ The samples were inoculated on sheep blood agar (DiMed, St. Paul, MN), eosin methylene blue agar (Baltimore Biological Laboratories, Cockeysville, MD), and chocolate agar (bioMerieux, Marcy l’ Etoile, France). All cultures were incubated under aerobic or anaerobic conditions as required and observed at 24, 48, or 72 hours. Individual pure bacterial colonies were transferred to two Vitek identification cards (GP for all Gram‐positive cocci and GN for all Gram‐negative bacilli [bioMerieux, Shanghai, China]) according to the manufacturer's instructions. Individual bacterial strains were identified according to standard bacteriological methods using a fully automated microbe identification analyser (VITEK 32; bioMerieux ATB, France).

### 2.4 Assessment of LPS levels in blood

A kinetic chromogenic limulus amoebocyte lysate (LAL) assay was used for plasma LPS quantification. Five milliliters of peripheral blood venous blood was drawn from the subject before antibiotic use, and the procedure was strictly performed according to the G^−^ bacteria LPS Detection Kit instructions (Kinetic Chromogenic LAL; Dynamiker Biotechnology Co, Ltd, Tianjin, China). The detection sensitivity of this reagent was 0.025 EU/mL, the detection range of blood samples was from 0.03 to 0.48 EU/mL, and patients with abnormal liver and kidney function tests were excluded.

### 2.5 Immunohistochemistry for LPS and GR‐α/β

After dehydration, antigen retrieval, and blocking endogenous peroxidase of paraffin sections, primary antibodies were incubated with the sections for 1 hour at room temperature: mouse anti‐*Escherichia coli* LPS monoclonal antibody (1:100, 1 mg/mL; ab35654; Abcam), rabbit anti‐GR‐α polyclonal antibody (1:100, 0.5 mg/mL; ab3580; Abcam), and rabbit anti‐GR‐β polyclonal antibody (1:100, 1 mg/mL; ab233165; Abcam). After washing, the sections were incubated with horseradish peroxidase‐labeled streptavidin‐biotin complex at 37°C in a humidified chamber for 30 minutes. The sections were washed and developed with diaminobenzidine and washed immediately with running water to terminate the reaction when brown particles appeared in the cytoplasm. Species‐matched and isotype‐matched antibodies were used as negative controls. Five fields were randomly selected and scored by two independent observers, as in the histologic study. The immunohistochemical results are expressed as the mean optical density.

### 2.6 Paraffin section immunofluorescence

Immunofluorescence was performed in accordance with previously described methods.^2^ After dehydration, antigen retrieval, and blocking endogenous peroxidase of paraffin sections, primary antibodies were incubated with the sections for 1 hour at room temperature: mouse anti‐*E. coli* LPS antibody (1:100, 1 mg/mL; ab35654; Abcam), rabbit anti‐cluster of differentiation 68 (1:100, 1 mg/mL; ab125047; Abcam), and rabbit anti‐myeloperoxidase antibody (1:100, 500 μL; ab9535; Abcam). A wash with 0.01M phosphate‐buffered saline (PBS) was conducted. Fluorescein isothiocyanate‐labeled goat anti‐rabbit immunoglobulin M (IgM) (1:100; LS‐C86590‐2000) or Luo Danming (rhodamine)‐labeled goat anti‐rat IgG secondary antibodies (1:100; LS‐C61649‐1000; both LifeSpan BioSciences, Inc, Seattle, WA) were added to the sections and incubated for 1 hour at room temperature. After a further wash with 0.01M PBS, the sections were counterstained with 4,6‐diamidino‐2‐phenylindole. Species‐matched and isotype‐matched antibodies were used as negative controls. Five fields were randomly selected and scored by two independent observers, as in the histologic study. Images were captured using an upright Olympus BX61 fluorescence microscope (Olympus Corporation, Tokyo, Japan).

### 2.7 Statistical analysis

For continuous variables, experimental results are expressed as the mean ± SD, and SPSS software (version 19.0; SPSS, Inc, Chicago, IL) was used to conduct statistical analyses. The Kruskal‐Wallis test was used to assess the significance of intergroup variability, and the Mann‐Whitney two‐tailed U test was used to assess significance for between‐group comparisons. The Spearman coefficient test was used to analyze the correlation between GR‐α/β and LPS in nasal tissues. The *χ* ^2^ test was used to analyze the difference in bacterial distribution in the nasal cavity and the LPS positivity in nasal tissue. *P* < .05 was considered to indicate a statistically significant result.

## 3 RESULTS

### 3.1 Neutrophilic and eosinophilic CRSwNP

CRSwNP was classified into noneosinophilic and eosinophilic CRSwNP according to a previously established standard.^1^ In this study, noneosinophilic CRSwNP applied to 63 cases, accounting for approximately 56% of the total, while 49 cases of eosinophilic CRSwNP accounted for approximately 44% of the total.

### 3.2 Bacterial colonization was not different between CRSwNP subjects and controls

The bacterial infection is an important factor for chronic inflammation of CRSwNP. In this study, 27 bacterial strains were isolated from 112 CRSwNP subjects, including 13 strains of G^+^ bacteria, 14 strains of G^−^ bacteria, and there were 20 cases without bacterial growth. There were 15 bacterial strains, including 7 strains of G^+^ bacteria, 8 strains of G^−^ bacteria, and 13 cases without bacterial growth in the controls. The main bacterial strains in the controls and CRSwNP subjects were all *Staphylococcus epidermidis*, coagulase‐negative staphylococci, *E. coli*, *Streptococcus pneumoniae*, and *Klebsiella pneumoniae*, and total G^+^ bacteria, G^−^ bacteria, and *E. coli* were not statistically significant between the two groups by the *χ* ^2^ test (Table 2).

**Table 2:** Results of bacterial culture

Groups	Control	CRSwNP	*P*
Samples	50	112	
Total bacteria	36 (72%)	92 (82.1%)	.150
G^+^ bacteria	23 (46%)	57 (50.9%)	.612
G^−^ bacteria	13 (26%)	35 (31.2%)	.578
*E. coli*	3 (6%)	11 (9.8%)	.553

*Note*: The positive rate of bacterial culture in nasal secretions of different groups was not different. The *χ* ^2^ test was used to analyze the difference of bacterial distribution in the nasal cavity. *P* < .05 was considered statistically significant.

Abbreviations: CRSwNP, chronic rhinosinusitis with nasal polyps; *E. coli*, *Escherichia coli*; G^−^, Gram‐negative bacteria; G^+^, Gram‐positive bacteria.

### 3.3 Increased LPS levels in peripheral blood of noneosinophilic CRSwNP subjects compared to those in peripheral blood from eosinophilic CRSwNP subjects and controls

**Figure 1:**
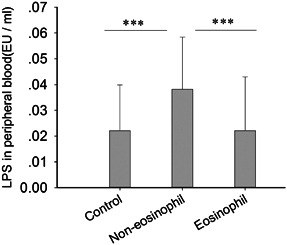
The expression level of lipopolysaccharide (LPS) in peripheral blood from different groups was significantly different. The Kruskal‐Wallis test was used to assess the significance of intergroup variability, and the Mann‐Whitney two‐tailed U test was used to assess significance for between‐group comparisons. *P* < .05 was considered statistically significant. *P < .05, **P < .01, ***P < .001

Although CRSwNP is a local inflammatory response, the levels of the G^−^ bacterial product LPS in the peripheral blood of CRSwNP subjects have not been detected. The detection cutoff of the LPS reagent was 0.025 EU/mL; levels could not be measured below this concentration, and the defect value was replaced with zero. The LPS levels in the peripheral blood of normal people were less than 0.053 EU/mL, but they were more than 0.109 EU/mL in patients with systemic infection by G^−^ bacteria. In this study, they were all below 0.109 EU/mL in all subjects. However, these values were significantly higher in the noneosinophilic CRSwNP group than in the control and eosinophilic CRSwNP groups (all *P* = .00) but not significantly different between the control and eosinophilic CRSwNP groups (*P* = .995) (Figure 1).

### 3.4 Increased LPS levels were positively correlated with upregulated GR‐β levels in noneosinophilic CRSwNP subjects compared to those in controls and eosinophilic CRSwNP subjects

**Figure 2:**
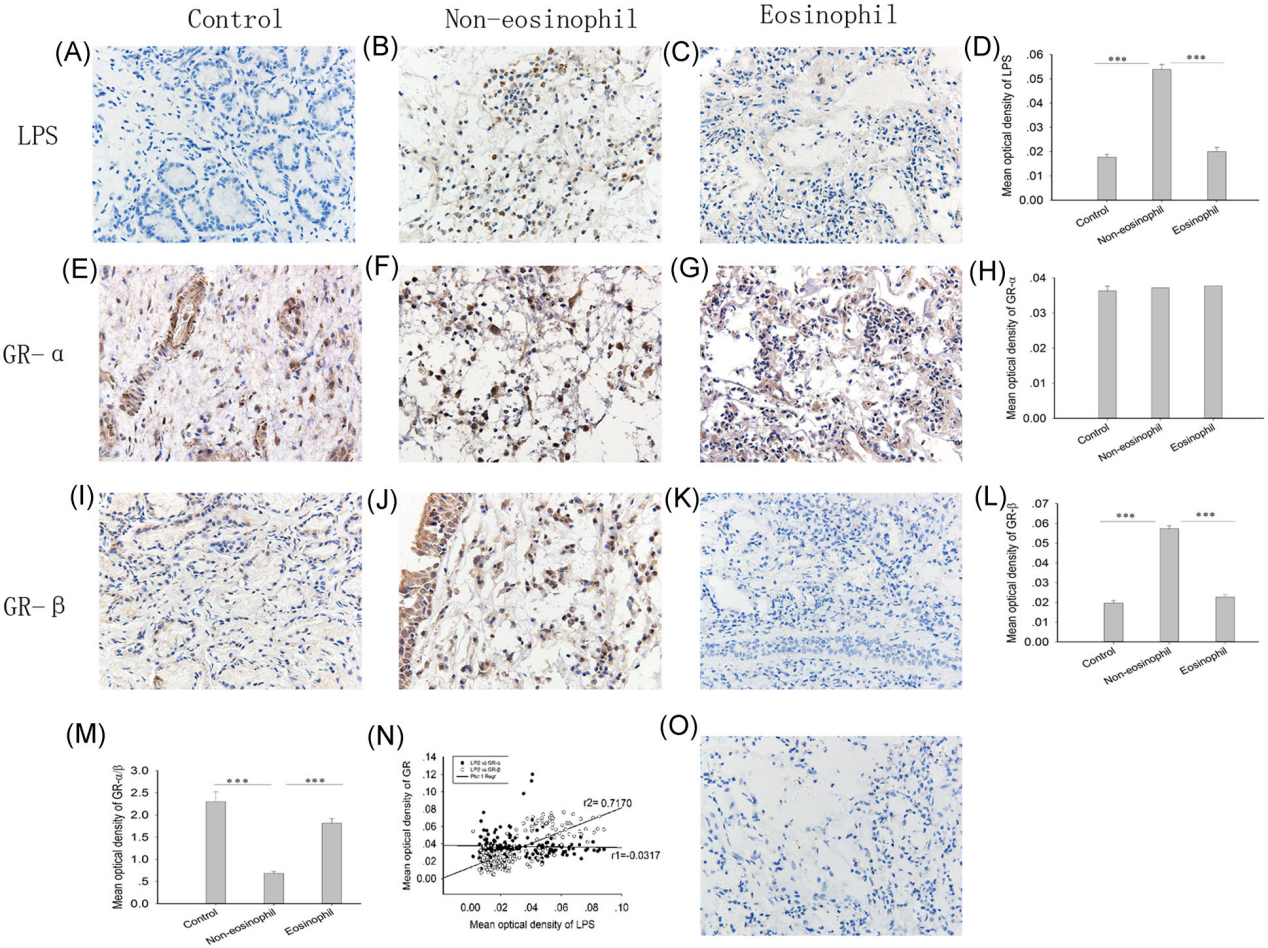
Immunohistochemistry for LPS, GR‐α, and GR‐β in controls and CRSwNP and their relationships. The data were expressed by the mean optical density, brown was representative of positive expression. Immunohistochemistry was used to analyze the expression of (A‐D) LPS, (E‐H) GR‐α, and (I‐L) GR‐β and the relative expression ratio of (M) GR‐α/β in the samples. N, The Spearman relationship of LPS with GR‐α or GR‐β. O, Species‐matched and isotype‐matched antibodies were used as negative controls. The Kruskal‐Wallis H‐test was used to assess significant intergroup variability. The Mann‐Whitney two‐tailed U test was used for between‐group comparison. The Spearman test was used to determine correlations. Original magnification was ×400. CRSwNP, chronic rhinosinusitis with nasal polyps; GR‐α, glucocorticoid receptor‐α; GR‐β, glucocorticoid receptor‐β; LPS, lipopolysaccharide. *P < .05, **P < .01, ***P < .001

Bacterial culture does not fully reflect the inflammation of the submucosa, and we further detected the expression of LPS in the nasal tissue. Approximately 10% of the controls were weakly positive for LPS, but 31 cases accounting for approximately 27.7% of the total CRSwNP subjects were positive, which was much higher than the rate in the controls. Furthermore, eosinophilic CRSwNP subjects had only weak positivity (16.3%) for LPS, while 23 subjects (36.5%) with noneosinophilic CRSwNP had moderate or strong LPS signals, which were significantly higher than those in eosinophilic CRSwNP subjects and the controls (all *P* = .00, Table 3 and Figure 2A‐D). The mean optical density of GR‐α was slightly increased in eosinophilic CRSwNP subjects, but there was no significant difference between all the groups (all *P* > .05, Figure 2E‐H). Compared to that in the control and eosinophilic groups, the mean optical density of GR‐β was significantly upregulated (all *P* = .00, Figure 2I‐L), but the ratio of GR‐α/GR‐β decreased in the noneosinophilic CRSwNP group (all *P* = .00, Figure 2M). The levels of LPS in CRSwNP subjects were positively correlated with GR‐β (*r* = .717, *P* = .00, Figure 2N) but not GR‐α expression levels (*r* = −.032, *P* = .689, Figure 2N).

**Table 3:** LPS positive rate in controls and patients with CRSwNP

Groups	N (number)	LPS^+^ rate	*P*
Controls	50	5 (10%)	.012^a^
CRSwNP	112	31 (27.7%)	
Noneosinophilic CRSwNP	63	23 (36.5%)	.018^b^
Eosinophilic CRSwNP	49	8 (16.3%)	

*Note*: The positive rate of LPS in the controls and patients with CRSwNP was significantly different. The *χ* ^2^ test was used to analyze the difference of the LPS positive in the nasal tissue. *P* < .05 was considered statistically significant.

Abbreviations: CRSwNP, chronic rhinosinusitis with nasal polyps; LPS, lipopolysaccharides.

^a^ Represents a comparison between the controls and CRSwNP.

^b^ Represents a comparison between noneosinophilic CRSwNP and eosinophilic CRSwNP.

### 3.5 Immunofluorescence for cells containing LPS

**Figure 3:**
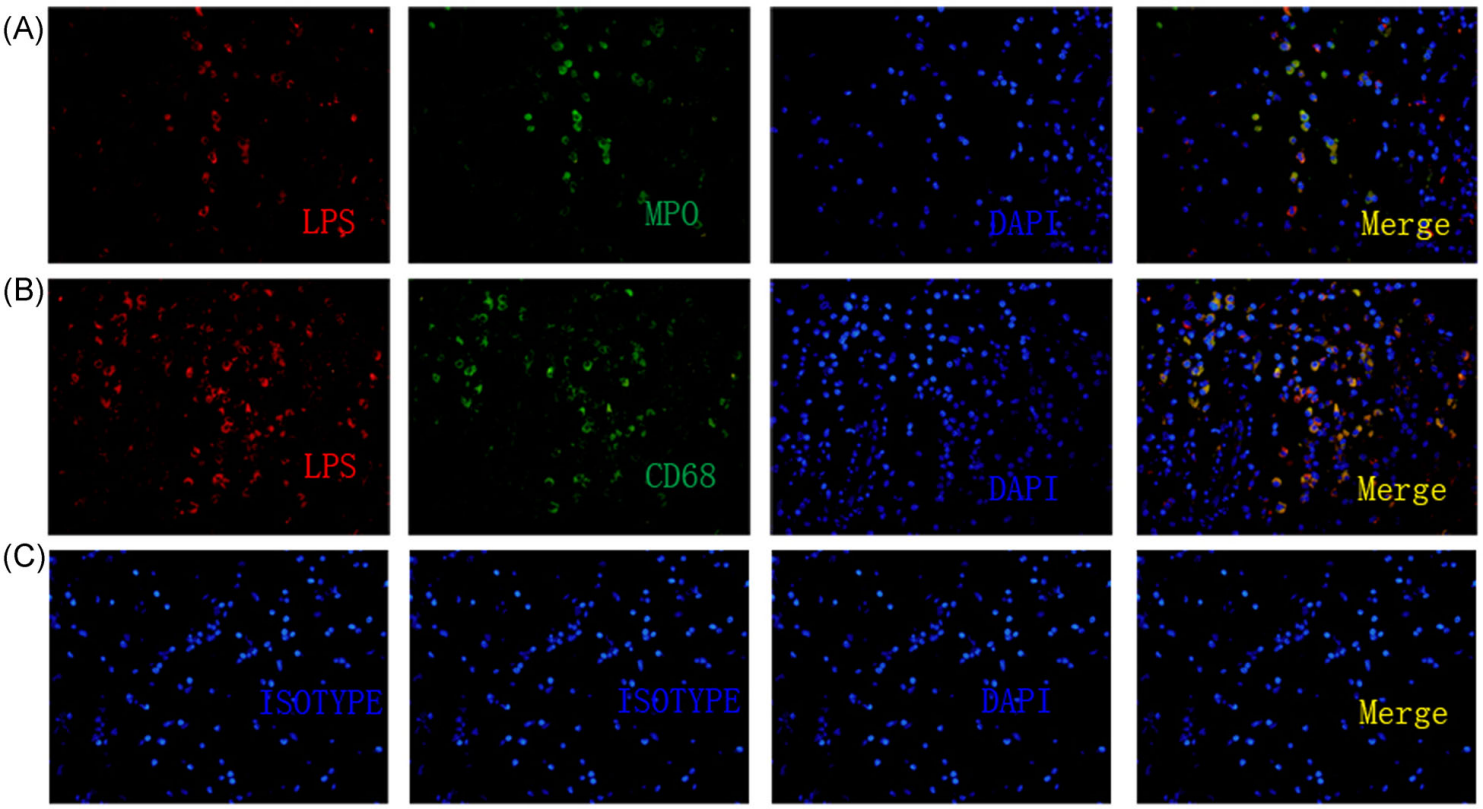
Detection of LPS in macrophages and neutrophils in CRSwNP. A and B, An immunofluorescence assay was performed with anti‐LPS mAb (A and B, red), anti‐CD68 Ab (A, green), and anti‐MPO Ab (B, green). Nuclei were counterstained with 4′, 6‐diamidino‐2‐phenylindole (DAPI, blue). C, Species‐matched and isotype‐matched antibodies were used as negative controls. The results were representative of 15 separate patients. Original magnification was ×400. CD68, cluster of differentiation 68; CRSwNP, chronic rhinosinusitis with nasal polyps; LPS, lipopolysaccharide; MPO, myeloperoxidase

LPS^+^ cells were found in the submucosal region in the control and CRSwNP groups. However, LPS detection levels were significantly elevated in the noneosinophilic CRSwNP group compared to those in the control and eosinophilic groups (all *P* = .00, Figure 2A‐D). We next proceeded to identify LPS‐containing cells in the nasal tissue of CRSwNP subjects through double‐labeling immunofluorescence. In addition to tissue and epithelial cells, the principal inflammatory cells containing LPS were neutrophils (15.8%, 5.4%‐28.2%) and macrophages (20.8%, 7.3%‐32.8%), and the latter had a higher ratio than the former (*P* = .00, Figure 3).

## 4 DISCUSSION

The present research found that the total positive rate of LPS in CRSwNP subjects was 27.7%, which was much higher than the 9.8% rate of *E. coli* in nasal secretions and was positively correlated with GR‐β expression levels. The principal inflammatory cells containing LPS were neutrophils and macrophages. However, the LPS levels in peripheral blood from CRSwNP subjects were lower than those in the recommended G^−^ infection standard. This result shows that LPS in CRSwNP subjects mainly plays a local inflammatory role and does not cause a systemic inflammatory response. Therefore, local production of LPS and possible resistance to GR may be one of the important pathogenic mechanisms of G^−^ bacteria in CRSwNP.

Refractory CRSwNP might be closely related to G^−^ bacteria, especially *E. coli*.^22^, ^23^, ^24^, ^25^ For example, Ba et al^22^ found that 70% of patients with CRSwNP in mainland China were characterized by neutrophilic inflammation and associated with greater G^−^ bacterial loads than controls. Contrary to the studies above, some studies suggest that the levels of G^−^ bacteria are not significantly different between patients with chronic rhinosinusitis and normal subjects,^14^ similar to our bacterial culture results. The variation among different research groups might be related to various research methods and different types of chronic rhinosinusitis. In contrast to G^−^ bacteria in nasal secretions, G^−^ bacteria, and LPS, combined with tissues and cells, could not be detected by bacterial culture. Moreover, Rom et al^14^ used nasal bacterial culture combined with tissue types to find that CRSwNP was not related to G^−^ or G^+^ bacteria in bacterial culture but to submucosal G^−^ infection. Consistent with the above results, our current LPS immunohistochemistry confirmed that the total positive rate of LPS in CRSwNP subjects was 27.7% and as high as 36.5% in noneosinophilic CRSwNP subjects, which was much higher than the 9.8% rate of *E. coli* culture. This result may be an important reason for the inconsistency in the results. Therefore, the simple method of nasal bacterial culture limits the understanding of the pathological mechanism of CRSwNP,^14^ and molecular diagnosis of submucosal bacterial clones and histopathological observation are more sensitive and diagnostic than simple bacterial culture.^26^, ^27^

LPS is considered to be a trigger for inflammatory responses. LPS activates inflammatory signaling pathways mainly through the classic Toll‐like receptor 4 (TLR4) receptor on the cell surface, stimulating the body to produce inflammatory factors, and leading to tissue remodeling.^28^, ^29^, ^30^ In addition, LPS can also promote inflammatory responses through other pathways. For example, nasal instillation of LPS in normal people can recruit neutrophils, inhibit the apoptosis of neutrophils, and further stimulate neutrophils to release the inflammatory mediator macrophage inflammatory protein‐1α.^31^ LPS can also directly stimulate macrophages to release a variety of additional inflammatory mediators, such as interleukin‐1 (IL‐1) and tumor necrosis factor, which can cause local inflammation to persist and eventually lead to tissue remodeling. Moreover, type one macrophages can also cause glucocorticoid resistance through interferon‐γ and TLR4.^32^ In addition to inflammatory cells, LPS also stimulates nasal epithelia to secrete inflammatory mediators, such as IL‐6, IL‐8, tumor necrosis factor‐α, and high‐mobility group box 1, to promote inflammatory responses or autophagy of epithelia.^30^, ^33^, ^34^ LPS can also promote fibroblasts to express vascular endothelial growth factor and induce the development of CRSwNP.^29^

LPS also exerts different effects on different microenvironments or with different doses. In contrast to high concentrations, low concentrations of LPS do not suppress but instead promote the allergic immune response.^16^, ^17^, ^18^, ^35^ In this study, the positive rate of LPS in eosinophilic CRSwNP subjects was only 16%, and it is generally weak, so we conclude that low levels of LPS may promote the occurrence and development of eosinophilic CRSwNP. High levels of LPS may be related to increased neutrophils in noneosinophilic CRSwNP. Nevertheless, we further found that the levels of LPS in peripheral blood were extremely low and almost in the normal range, and there was no significant difference between the controls and CRSwNP subjects. Unlike acute rhinosinusitis,^13^, ^21^ CRSwNP is a chronic inflammatory reaction in the nasal cavity and sinuses, and its main symptoms include discomfort, such as nasal congestion, runny nose, dizziness, and olfactory disorders, but not systemic symptoms, such as fever and systemic inflammation, and the number of white blood cells in the peripheral blood is within the normal range.^21^ The present study supports that LPS plays only a local and pathogenic role in CRSwNP.

Next, we further observed that the major LPS^+^ inflammatory cells were neutrophils and macrophages by double‐labeling immunofluorescence, and the LPS levels of macrophages were much higher than those of neutrophils. Neutrophils and macrophages are the two main types of phagocytes. Upon phagocytosing microorganisms, they rapidly activate intracellular enzyme systems to degrade microorganisms into small molecules and harmless substances. Neutrophils have a short life span, and their numbers soon decrease after antibiotic treatment. However, macrophages have a long life span and are relatively difficult to recruit when entering local tissues from peripheral blood.^1^, ^4^ For example, in animal models, when LPS was dropped into the trachea of rats, the neutrophil numbers in bronchoalveolar lavage fluids increased only for a few days and then began to decline, while the number of macrophages increased and was maintained for approximately 40 days.^36^ Therefore, the current research suggests that LPS may mediate a persistent inflammatory response mainly through macrophages in CRSwNP. This hypothesis has also been confirmed in animal models.^19^

As glucocorticoids are the first‐line medical therapy, glucocorticoid resistance is very important for the recurrence of CRSwNP. GRs are divided into two types: GR‐α and GR‐β. GR‐α is a sensitive receptor for glucocorticoids but is antagonized by GR‐β. Increased GR‐β or GR‐α dysfunction or their imbalanced proportion is related to glucocorticoid resistance.^6^, ^9^, ^11^, ^12^, ^37^, ^38^ In Asian countries, such as China, CRSwNP is predominantly noneosinophilic,^1^ and refractory CRSwNP is commonly related to G^−^ bacterial infections.^7^, ^13^, ^14^ Wen et al^10^ found that glucocorticoids had a poorer effect on noneosinophilic CRSwNP than eosinophilic CRSwNP because of increased neutrophil levels. Consistent with the above studies, we further found that the expression of GR‐β and LPS content in noneosinophilic CRSwNP subjects were significantly elevated compared to those in eosinophilic CRSwNP subjects and the controls. Moreover, LPS levels were significantly and positively correlated with GR‐β levels. Nevertheless, GR‐α expression levels were only slightly upregulated in the eosinophilic CRSwNP group but not significantly different among all the groups. Our results suggest that LPS may cause glucocorticoid resistance in CRSwNP in vivo. Laura et al^39^ analyzed the molecular mechanism of LPS on glucocorticoid resistance in CRSwNP in vitro, including inhibiting dexamethasone‐induced glucocorticoid GR‐α nuclear translocation and zipper protein expression and reducing the inhibitory inflammatory effect of dexamethasone on mediators. LPS could also induce monocytes to secrete macrophage migration inhibitory factors, downregulating GR function.^40^, ^41^, ^42^ In addition, LPS downregulated MUC1‐CT/GR‐α complex expression and upregulated GR‐β on neutrophils, leading to a decreased effect of dexamethasone.^43^, ^44^ These studies support that LPS and induced neutrophils can cause glucocorticoid resistance in vitro. Inconsistent with our research, Watanabe et al^45^ found that GR‐α expression in CRSwNP was mainly distributed in inflammatory cells, such as macrophages and lymphocytes, and was significantly upregulated compared to that in the controls but decreased after glucocorticoid treatment. However, GR‐β expression in CRSwNP subjects was not different from that in normal controls or was not affected by glucocorticoid therapy. The possible explanations for these inconsistent results from different research teams are as follows. First, CRSwNP was not further classified into neutrophilic and eosinophilic types according to the degree of infiltrative eosinophils.^1^, ^3^, ^4^, ^10^, ^46^ Second, CRSwNP was not divided into seromucinous, fibroinflammatory, and edematous types according to the degree of tissue remodeling.^2^ Third, different research teams used various research methods, such as polymerase chain reaction, immunohistochemistry, immunofluorescence, and Western blot.^2^, ^37^ Fourth, there were activated and inactive forms or cross‐reactivity of GRs; for example, the messenger RNA and protein expression and distribution of GR‐β in the bronchial epithelium were not different among different groups but were significantly different by immunohistochemistry because of cross‐reactivity.^37^

In this study, we chose *E. coli*‐derived LPS as the research object because the bacterial culture showed that the highest positive rate of G^−^ bacteria was *E. coli*, which was consistent with previous research.^22^ However, the LPS antibody was derived from a partial fragment of *E. coli*, and the molecular structure of LPS from different G^−^ sources is not completely consistent; therefore, the use of a single source of LPS in this study might reduce the total positive rate of LPS in CRSwNP. In addition, we showed that LPS levels in the peripheral blood of patients with CRSwNP were lower than the G^−^ infection threshold of 0.109 EU/mL, which met the characteristics of CRSwNP characterized by local lesions. Although the present study did not study the expression of TLR4 in CRSwNP, previous studies have shown that TLR4 expression is upregulated and related to inflammatory processes in CRSwNP.^47^, ^48^

In conclusion, the present study demonstrates that there is abundant LPS in CRSwNP, especially for noneosinophilic CRSwNP, which is much higher than the positive rate of *E. coli* in nasal secretions. Moreover, LPS levels are positively correlated with GR‐β expression in CRSwNP. Nevertheless, LPS levels in the peripheral blood of patients with CRSwNP are lower than infection standard levels. Therefore, local increased LPS content can cause mucosal inflammation to persist and eventually lead to tissue remodeling and the development of CRSwNP. We conclude that LPS production and inducing possible resistance to glucocorticoids may be one of the important pathogenic mechanisms of G^−^ bacteria in CRSwNP.

## CONFLICT OF INTERESTS

The authors declare that there are no conflict of interests.

## AUTHOR CONTRIBUTIONS

S‐BW contributed to design the study, carried out data analysis, and wrote the manuscript. K‐SZ helped for immunohistochemistry and its quantification. BZ contributed to the design of study and immunohistochemistry. LC helped for assessment of lipopolysaccharide levels in blood assays and reviewed the manuscript. X‐YZ helped for bacterial culture. S‐MC contributed to design the study and reviewed the data and manuscript.

## Supporting information

Supporting information

## Data Availability

The data that support the findings of this study are available from the corresponding author upon reasonable request.
