# Supplementary material for: Increased lipopolysaccharide content is positively correlated with glucocorticoid receptor‐beta expression in chronic rhinosinusitis with nasal polyps
Source: Immun Inflamm Dis. 2020 Sep 1;8(4):605–14. doi: 10.1002/iid3.346 (PMC7654414; doi:10.1002/iid3.346)

SUPPORTING INFORMATION

Additional supporting information may be found in the online version of this article at the publisher’s web-site.

Figure S1. Morphological staining of the two types of tissues from patients with CRSwNP. H&E stains the cytoplasm of eosinophilic cells red. H&E stain enable CRSwNP to be divided into two types, namely the eosinophilic(A-B) and non-eosinophilic(C-D) types. Original magnification, 400x. H&E, hematoxylin and eosin; CRSwNP, chronic rhinositus with nasal polyps.


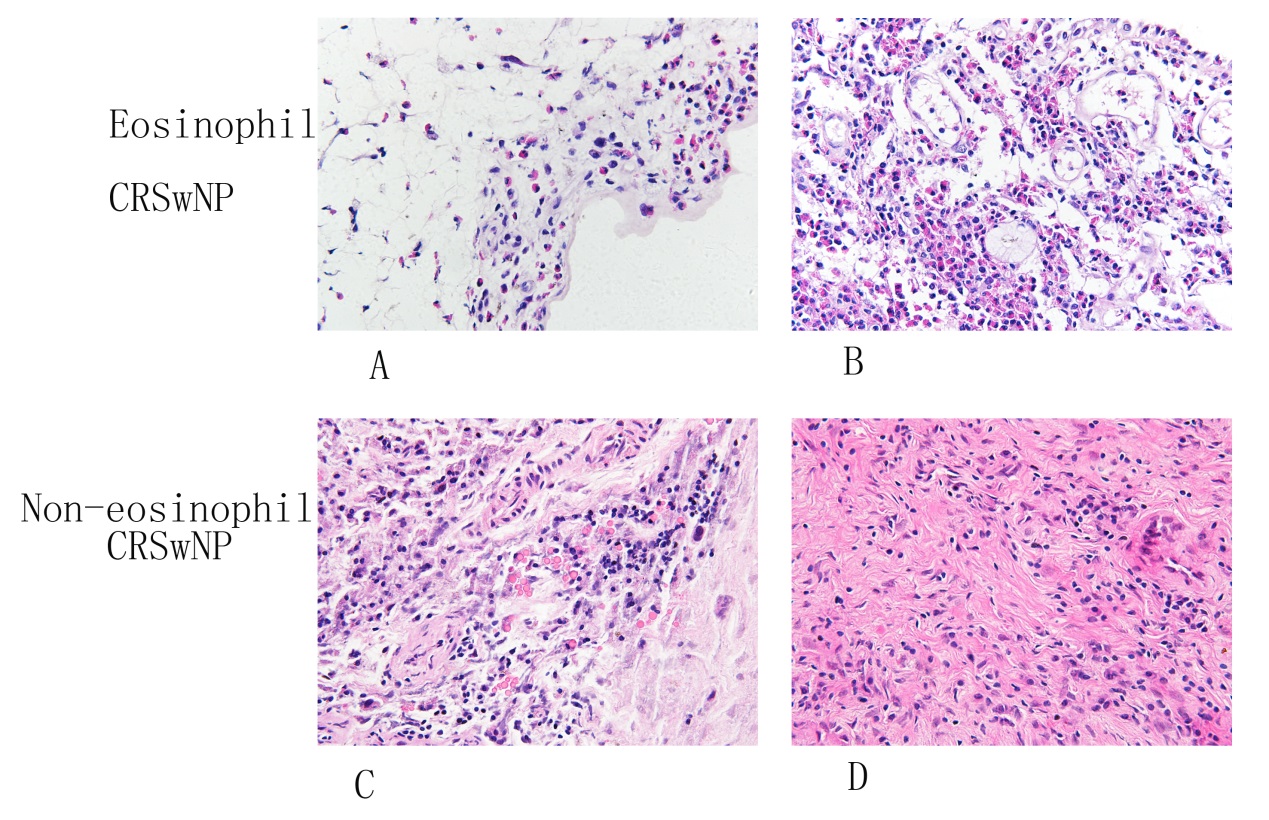

Supplement: Supplementary file 1 — Supporting information [file IID3-8-605-s001.docx]
